# Patient Mobility Support for Indoor Non-Directed Optical Body Area Networks

**DOI:** 10.3390/s19102297

**Published:** 2019-05-18

**Authors:** Durai Rajan Dhatchayeny, Sudhanshu Arya, Yeon Ho Chung

**Affiliations:** Department of Information and Communications Engineering, Pukyong National University, Busan 48513, Korea; enithendral@gmail.com (D.R.D.); sudhanshu.arya27@gmail.com (S.A.)

**Keywords:** optical communications, light-emitting diodes, mobility, diversity

## Abstract

In this paper, a patient mobility support scheme for indoor non-directed optical body area networks (OBAN) is presented. The OBAN is an optical healthcare system where medical sensors are installed on various parts of the patient’s body and are connected to an optical coordinator for transmitting the physiological signals via optical wireless links. In the proposed scheme, a white light-emitting diode (LED) was employed as the optical coordinator that was mounted on the patient body, while a photodetector (PD) was used as the receiver installed at the ceiling. We considered three practical mobility scenarios in terms of the location of the coordinator: (i) Shoulder, (ii) wrist, and (iii) both shoulder and wrist. The analytical channel model for multiple reflections in a non-directed OBAN was developed and validated in the form of simulations. In addition, experiments were carried out to verify the effectiveness of the proposed mobility scheme. It was found that the third scenario (shoulder and wrist) performed best, showing a bit error rate (BER) of 1.2 × 10^−6^ at a distance of 1.25 m. The experimental results demonstrated that the proposed mobility support scheme in the OBAN added an additional degree of freedom to patients with reliable performances.

## 1. Introduction

Rapid progression of healthcare technologies has been providing numerous benefits for human welfare and has improved the quality of health services provided to patients. These technologies aid doctors and medical professionals in monitoring patient health status in an incessant manner, and thus offer early detection, diagnosis, and monitoring of diseases that facilitate patients with optimal medical assistance [1,2].

One such promising candidate for healthcare monitoring is wireless body area network (WBAN). Generally, WBAN is based on radio frequency (RF) technologies having wearable sensor devices mounted on the patient’s body that are capable of various functions such as sensing, processing, and communicating physiological signals [3,4]. Despite advantages of RF-based WBAN, such as flexibility and mobility, performance is limited by electromagnetic interference (EMI) and may result in erroneous information transmission. EMI can affect various healthcare devices such as fetal monitors, infusion pumps, syringe pumps, and ventilators [5,6]. Moreover, the RF transmission of some biomedical signals, such as electroencephalography (EEG), electrocardiography (ECG) and photoplethysmography (PPG), suffers from a lack of precision due to interference, noise, and multi-frequency characteristics of various signals [7].

An alternative method is to employ optical wireless communication (OWC) technology for healthcare applications. The OWC link offers EMI-free data transmission along with minimizing RF health impairment effects around the patient’s vicinity and can be employed inside sensitive environments such as an intensive care unit (ICU) where RF-based devices are prohibited. In addition, optical wireless based systems are license free, compact, low cost, and have a high level of security [8,9].

Recently, an infrared (IR) based patient mobility support scheme was proposed in a diffuse optical configuration [10]. The theoretical analysis of an on-body system with mobility support (both the IR emitter and the receiver placed on the patient body) was conducted using an optical code division multiple access (OCDMA) strategy over diffuse IR optical links [11]. Specifically, the power consumption with three transmitting nodes placed on the body was analyzed. However, there is a trade-off between the performance and the complexity if more medical sensors are employed on the body. Moreover, the analysis was validated only by the statistical approach without any practical or realistic demonstrations.

To transmit some biomedical data using visible light communication (VLC), interesting studies were reported in the literature. A biomedical signal, such as an EEG, was transmitted via on-off keying (OOK) modulation using selection combining scheme [12]. It was experimentally demonstrated that this scheme offers a reliable transmission of the biomedical signal using the VLC. Unfortunately, the work did not consider the important issue of mobility support. A multi-patient vital signs data transmission using VLC was also presented. It employed orthogonal codes for separating each patient’s data [13].

Like WBAN, patients are likely to move around in a room where an optical body area network (OBAN) (coordinator on the body and receiver at the ceiling) is established. Thus, the patient mobility support is considered one of the most essential and foremost aspects of hospitalized patients. The present study is motivated by the fact that there has been no patient mobility support using VLC links in an OBAN system in the literature. We developed a mobility scheme in an indoor OBAN. To support mobility in an OBAN brings new challenges and issues to the evolution of networks as it encompasses transmitter mobility rather than receiver mobility. The most convenient and performing configuration in OWC is the line-of-sight (LOS) link in which the transmitter and receiver are directed towards each other. The non-LOS (non-directed) mechanism is commonly employed in mobile-receiver based indoor optical wireless communication systems. This configuration is considered the most flexible in the literature [14]. Considering the variations in human body movements in an OBAN environment, it is not always possible to establish a perfect link between the transmitter and the receiver. In this view, we considered non-directed optical links. The OBAN system consists of body sensors fixed on various parts of the body for monitoring the physiological parameters. These sensors are directly connected to an optical coordinator. The optical coordinator is composed of a white light-emitting diode (LED) which collects physiological signals from the sensors and transmits the data via an optical link. The photodetector (PD) is used as the receiver installed on the ceiling. In addition, we focused on non-directed optical links and developed an analytical model for multiple reflections. In the proposed mobility support scheme, we considered three mobility scenarios. The first scenario is that the coordinator is fixed at the shoulder and the second is that the coordinator is placed at the wrist of the patient body. The third scenario involves the coordinators placed at both the wrist and the shoulder. This scenario can be viewed as the transmit diversity to exploit for the mobility-supported OBAN. The performance of the proposed mobility model is evaluated in terms of bit error rate (BER) and experiments are conducted indoors to verify the effectiveness.

The contributions of the present study are listed below:

- An effective and convenient mobility support model is designed;
- Analytical expressions are newly derived for the VLC-based mobility support model;
- Simulations and experimental validations are performed;
- Multiple LEDs placed on body are employed for both data transmission and transmit diversity without causing any interference;
- Comparative study on mobility support scheme is presented.

The rest of the paper is organized as follows. In Section 2, the description of the OBAN system and the mobility model is provided. The channel model is presented in Section 3. Section 4 details the simulation results. Section 5 describes the experiments and validates the results. Conclusions are drawn in Section 6.

## 2. Proposed System Overview

**Figure 1:**
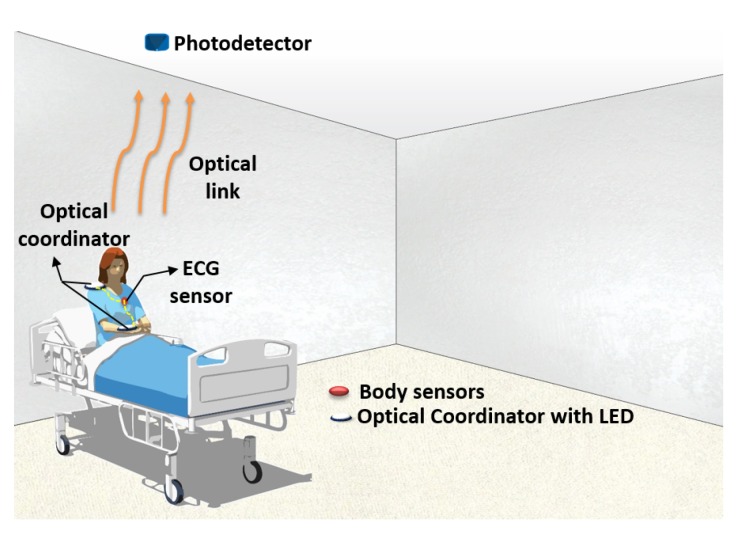
Proposed optical body area network (OBAN) system.

The proposed OBAN system architecture is shown in Figure 1. The OBAN system consists of sensors on various parts of the body for monitoring physiological signals. All the sensor nodes are directly connected to the optical coordinator via wires. The optical coordinator is composed of a LED which transmits the data via an optical link. The optical coordinator is fixed at both the wrist and the shoulder of the patient body. The PD is installed on the ceiling to receive the data.

**Figure 2:**
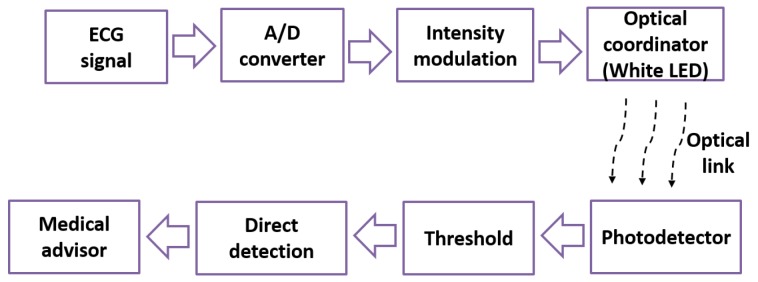
Block diagram of the proposed system.

Figure 2 illustrates the block diagram of the proposed system. We used the ECG as one of the biomedical signals for transmission. The detected ECG signal was sampled, quantized, and converted to binary data using an analog to digital (A/D) converter. The binary data is intensity modulated and then transmitted over the optical link using a single white LED as shown in Figure 2. At the receiver, a PD was utilized to convert the light intensity values of the received signal into a corresponding electrical signal. An adaptive threshold was used to retrieve the information more precisely [15]. Finally, the recovered data was evaluated by the medical advisor.

### Proposed Mobility Model

We considered the OBAN mobility scenarios where a patient was equipped with an ECG monitoring sensor and it was connected to an optical coordinator. The optical coordinator was composed of a white LED emitting an optical power of around 20 mW. The coordinator was fixed at the wrist and shoulder of the patient body. Due to the roughness of typical indoor environment surfaces that is characterized by a reflectivity parameter ρ, it was possible to consider diffuse reflections when the beam hit the surface. Then, the reflected signals were collected at the receiver fixed at the center of the ceiling oriented towards the floor. The receiver consisted of a PD with a large field of view (FOV) of 70° and a minimal irradiance of 1 μW/cm^2^. For the experimental setup, we considered room dimensions of 5 m × 5 m × 3 m.

**Figure 3:**
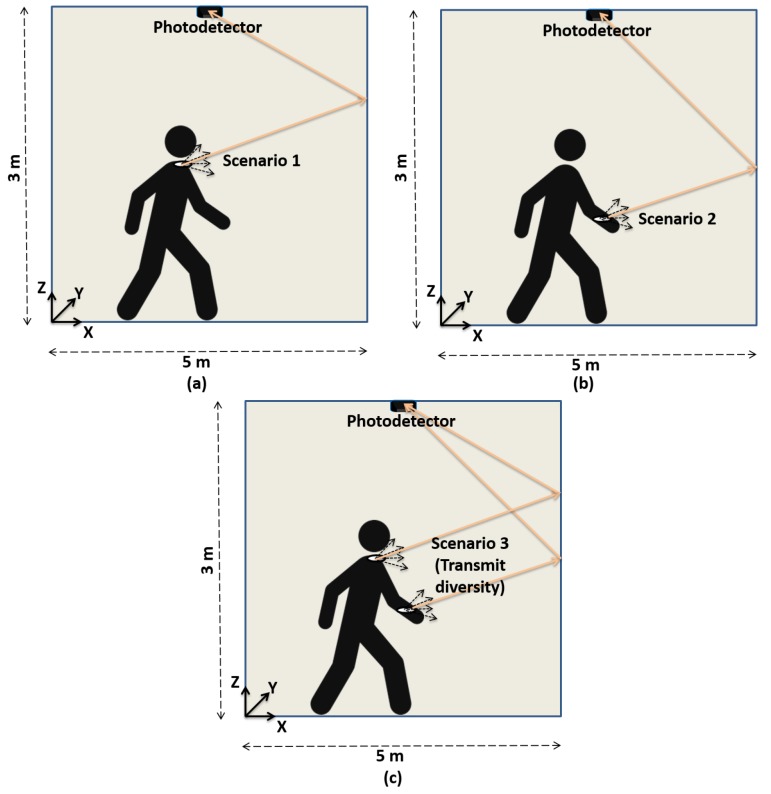
Mobility support with the coordinator placed on the body and the receiver on the ceiling. (**a**) Coordinator at shoulder. (**b**) Coordinator at wrist. (**c**) Coordinator at both shoulder and wrist.

Our aim is to provide mobility support via the non-directed optical links to transmit physiological signals of a moving patient. In this context, we considered three mobility scenarios. The first scenario involved the optical coordinator fixed at the shoulder of the patient body as shown in Figure 3a. The transmitter moves at an uniform distribution from 0 to 5 m along a *x* and *y* axis and the height is fixed at 1.5 m. Figure 3b describes the second scenario in which the optical coordinator was fixed at the wrist part of the body. In this scenario, the mobility was supported for all three axes along with the variations in focal angle $\alpha_{T_{x}}$ of the LED. Figure 3c shows the third scenario. It is the transmit diversity scenario in which the patient is equipped with two transmitters, i.e., one at the wrist and the other at the shoulder part of the body to perform the transmission of the monitored health care data. The transmit diversity exploiting two optical channels was used to mitigate the adverse effect of the patient movements, thereby achieving high diversity gains.

## 3. Channel Model

For the optical data transmission, we evaluated the optical wireless channel behavior with respect to the transmitter and the receiver. For indoor applications, the channel can be modeled as a linear time-invariant (LTI) system [16]. In non-directed optical links, the received optical power consists of the power from the directed and non-directed optical links over the surface environment. The received signal thus depends on the incident optical power and the PD responsivity. Hence, the received signal can be written as:

(1) Y=Sx(t)∗h(t)+n(t)

where *S* is the PD responsivity, *x*(*t*) is the transmitted signal, *h*(*t*) is the channel impulse response, *n*(*t*) is the additive white Gaussian noise (AWGN) generated from the ambient light sources, and ∗ denotes the convolution [16].

The channel path loss is given as:

(2) $$H\left(0\right)=\int_{-\infty }^{\infty }h\left(t\right)dt$$

where *h*(*t*) is the channel impulse response.

The reflection coefficient of the wall surface is set to ρ = 0.8, considering a wavelength of 875 nm [16]. The receiver FOV along with the transmitter and receiver orientation was also considered in analyzing the channel response. With the transmitter mobility, the channel static gain *H*(0) randomly varies due to the random transmitter position. To determine the optical gain probability density function (PDF), the experiment was conducted with different transmitter positions distributed uniformly over a 2-dimensional plane in the room. The source was supposed to be fixed at a height of 1 m above the floor. From the simulations considering a uniform distribution of (x, y) transmitter coordinates in the room, we estimated the PDF of the static channel gain. The received power is given as:

(3) $$P_{R}=H(0)P_{t}$$

where $P_{t}$ is the average transmitted optical power. Using Equation (3), the received power at varying distances was measured and the indoor channel path loss characteristics was determined.

**Figure 4:**
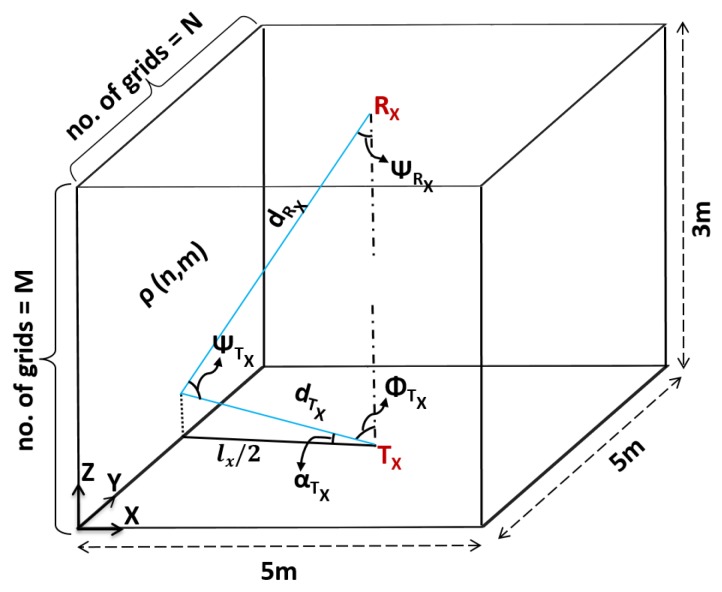
Analysis of the multiple reflected signals.

Based on the above observations, we took into account distance and angle for multiple reflection points in a non-directed optical link. In room dimensions of 5 m × 5 m × 3 m, each reflection point has a reflection area Δ*A* with an approximate distance between two reflection point sources being 1 cm. Using the illustration shown in Figure 4, we could derive the indoor channel response for multiple reflections.

For diffused reflections, the optical channel can be described by two components: (a) Each element of the surface with the area of Δ*A* considered as the receiver and (b) then each element considered as a point source that re-emits the light scaled by reflectivity ρ. The indoor channel path loss for non-directed optical links for multiple reflections from a single wall can be expressed as:

(4) $$H_{NLOS}\left(T_{x},R_{x},\rho \right)=\sum_{m=1}^{M}\sum_{n=1}^{N}\frac{\left(m_{l}+1\right)\rho \left(n,m\right)A_{r}\Delta A}{2\pi {\left[d_{T_{x},\rho }\left(n,m\right)\right]}^{2}{\left[d_{R_{x},\rho }\left(n,m\right)\right]}^{2}}\cos\left(\phi_{T_{x}}\left(n,m\right)\right)\cos\left(\psi_{T_{x}}\left(n,m\right)\right)\cos\left(\psi_{R_{x}}\left(n,m\right)\right)$$

where $T_{x}$ is the transmitter, $R_{x}$ is the receiver, $m_{l}$ is the Lambertian order, and $A_{r}$ is the area of the receiver. Each wall was divided into N×M reflection points where *N* and *M* are the number of divisions on the Y- and Z- axis. ρ represents the reflective property of the wall surface. Each point on the wall surface has a different distance and angle between the transmitter and receiver. The propagation distance between the transmitter and receiver is expressed as:

(5) $$l_{x}\left(n\right)=\sqrt{{\left(n-\frac{1}{2}\right)}^{2}+{\left(\frac{l_{x}}{2}\right)}^{2}}$$

where n represents the *n*th point along the y-axis on the wall surface.

The distance between the transmitter $T_{x}$ and the (n,m)th reflection point on the wall is given by:

(6) $$d_{T_{x},\rho }\left(n,m\right)=\sqrt{{\left(m-\frac{1}{2}\right)}^{2}+{l_{x}\left(n\right)}^{2}}$$

The focal angle, $\alpha_{T_{x}}\left(n,m\right)$, is the angle between $l_{x}\left(n\right)$ and $d_{T_{x},\rho }\left(n,m\right)$ and it is derived as:

(7) $$\alpha_{T_{x}}\left(n,m\right)=\tan^{-1}\left(\frac{2m-1}{2l_{x}\left(n\right)}\right)$$

The angle of irradiance, $\phi_{T_{x}}\left(n,m\right)$, can then be calculated as:

(8) $$\phi_{T_{x}}\left(n,m\right)=\frac{\pi }{2}-\alpha_{T_{x}}$$

The distance between the (n,m)th reflection point on the wall and the receiver $R_{x}$ is given by:

(9) $$d_{R_{x},\rho }\left(n,m\right)=\sqrt{{l_{x}\left(n\right)}^{2}+{\left(h-m+\frac{1}{2}\right)}^{2}}$$

The angle of incidence, $\psi_{R_{X}}\left(n,m\right)$, can be obtained as:

(10) $$\psi_{R_{x}}\left(n,m\right)=\cos^{-1}\left(\frac{2h-2m+1}{2d_{R_{x},\rho }\left(n,m\right)}\right)$$

The angle $\psi_{T_{X}}\left(n,m\right)$ is then calculated as:

(11) $$\psi_{T_{x}}\left(n,m\right)=180-\psi_{R_{x}}\left(n,m\right)-\phi_{T_{x}}\left(n,m\right)$$

While substituting Equations (6), (8), (9), (10), and (11) in Equation (4), the channel response for the multiple reflections can be obtained.

## 4. Simulation Results

**Figure 5:**
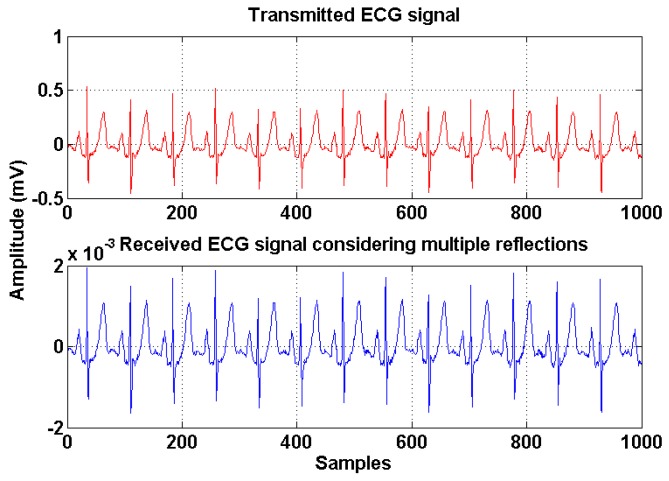
Transmitted and received electrocardiography (ECG) signal in non-directed optical links.

The validation of the analytical expressions was performed by Matlab simulations and the parameters are described in Table 1. Figure 5 shows the transmitted and received ECG signals in the non-directed optical links with all the reflections considered. The ECG biomedical signals are obtained from PhysioNet database [17]. The received signals from the non-directed links consist of various components arriving from different paths with different path lengths. This phenomenon results in the lengthening of the received signal.

**Figure 6:**
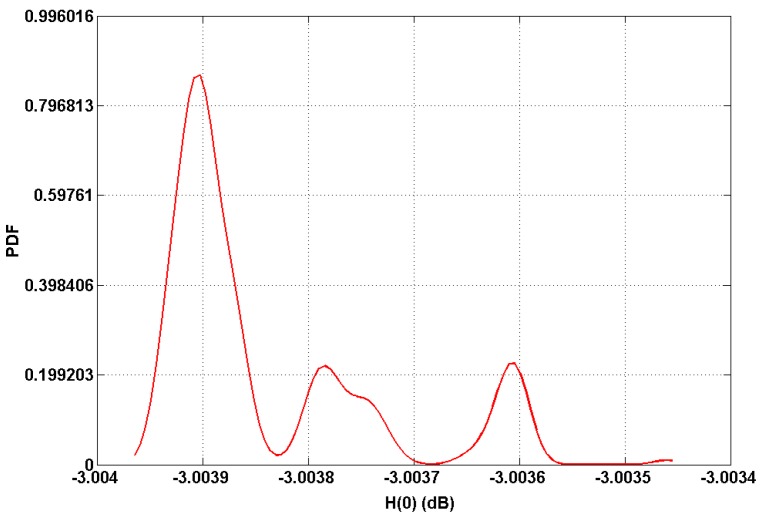
Probability density function (PDF) of optical channel gain.

Based on the analysis of the indoor channel model, we plotted the PDF of the static gain *H*(0) values in dB as shown in Figure 6. As expected, it can be seen that the gain reaches its highest value (around −3.0036 and −3.0038 dB) corresponding to LOS paths, whereas the maximum gain reaches at −3.0039 dB from the non-directed configurations.

**Figure 7:**
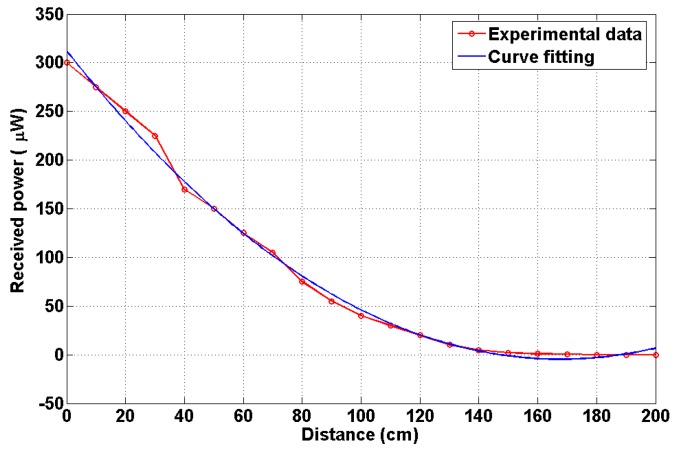
Channel path loss characteristics.

Figure 7 represents the indoor channel path loss characteristics. The path loss is obtained using a curve fitting technique with the second order polynomial function. It is noted that the fitted and experiment values are in good agreement. The received power decreases when the distance between the transmitter and the receiver increases.

**Figure 8:**
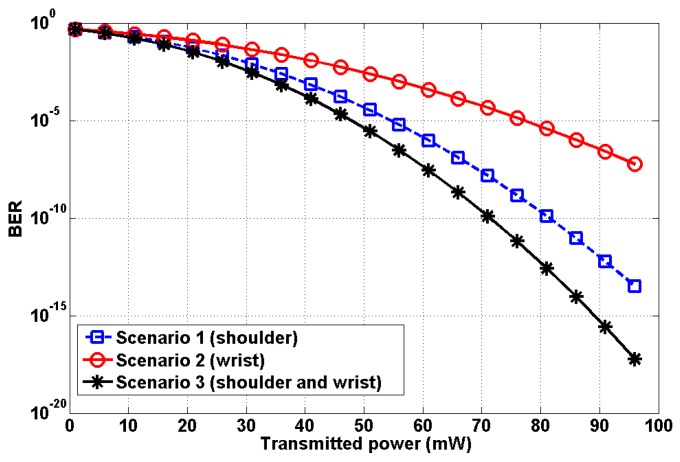
Performance analysis relative to transmit power.

We plotted the BER relative to the transmitted power $P_{t}$. Figure 8 shows the result. For the third scenario, synchronous transmission was performed with no interference between the two transmitters. The BER performance of Scenario 3 outperforms Scenarios 1 and 2.

In addition, we performed further analysis in order to highlight the superiority of the proposed model. Specifically, we considered the following three schemes:

- Proposed scheme: VLC link, OOK modulation, LED (on body), PD (ceiling), 2 nodes (shoulder and wrist);
- IR-OCDMA scheme [11]: IR link, OCDMA, IR-LED (on body), IR-PD (on body), 5 nodes;
- VLC-OCDMA scheme: VLC link, OCDMA, LED (on body), PD (ceiling), 2 nodes (shoulder and wrist).

**Figure 9:**
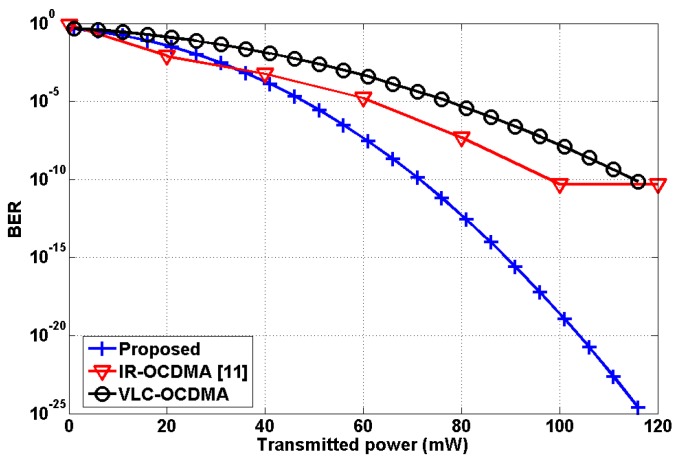
Performance comparison relative to transmit power.

It should be noted that the VLC-OCDMA scheme was not reported in the literature, but it is newly created for comparison purposes. The performance comparison is depicted in Figure 9. The proposed mobility support scheme is found to be superior in performance to the other two considered schemes as it achieves very low BER even by employing a fewer number of transmitting nodes, i.e., 2. In addition, it can be seen that in the proposed scheme the BER decreases with increasing transmitting power. Meanwhile, the error performance of the VLC-OCDMA is far poorer than the proposed scheme. The IR-OCDMA [11] produces relatively much higher BERs as the transmit power increases, compared with the proposed scheme. Moreover, the irreducible error performance occurs at the transmit power of 100 mW.

Some numerical comparative analysis is presented in Table 2. This comparison highlights the superiority of the proposed scheme in terms of achievable BER and number of nodes employed. In addition, the proposed scheme offers a very convenient and practical mobility support model compared with previous schemes reported in the literature.

As shown by the results, it is worth noting that the performance of the proposed mobility scheme can be further improved either by increasing the transmit power or by employing sophisticated modulation techniques such as pulse position modulation (PPM). The proposed VLC-OBAN mobility model has advantages of reliable data transmission with no interference between nodes. It is also relatively easy to design and implement practically.

## 5. Experiments and Results

### 5.1. Experiments

**Figure 10:**
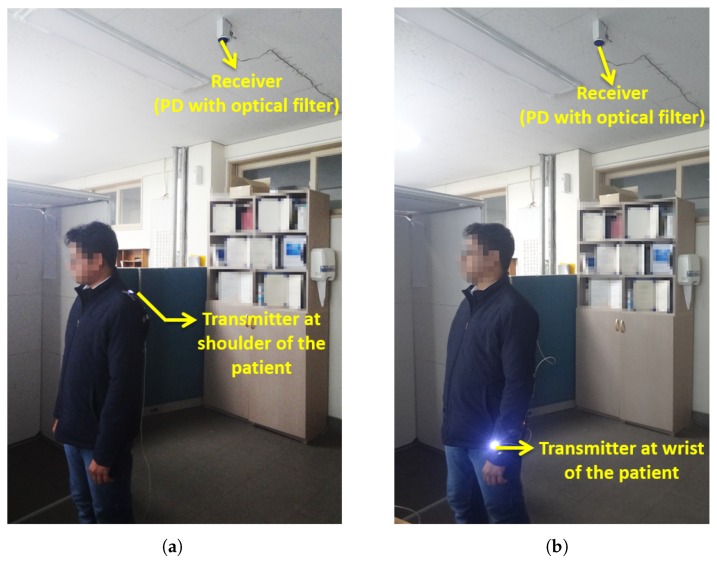
Experiments: (**a**) Mobility scenario 1. (**b**) Mobility scenario 2.

Experiments were conducted indoors to verify the proposed OBAN mobility model. A white LED was used as the transmitter and a PD with an optical filter was employed as the receiver. Table 3 shows the experiment parameters. The patient was assumed to move at an average speed of 0.5 m/s regularly throughout the experiment. As discussed previously, the experiments were carried out in three scenarios. In the first scenario, the optical coordinator was fixed at the shoulder of the patient body as shown in Figure 10a. The patient with the transmitter moves at an uniform distribution from 0 to 5 m along the x-axis, from 0 to 5 m along the y-axis and the transmitter height was fixed at 1.5 m above the floor. We also performed experiments by varying the transmitter height.

The second scenario involves the optical coordinator fixed at the wrist part of the body as shown in Figure 10b. The transmitter height was fixed at 0.5 m above the floor. In this scenario, mobility was supported for all three axes and the height of the transmitter varied uniformly from 0.5 to 1.5 m above the floor. The various human body movements led to the variations of the distance between the transmitter and receiver.

In the two scenarios, there were chances for the light to be blocked by the patient due to different postures of the body. This obstruction could be alleviated by the transmit diversity (scenario 3). We employed the two transmitters with identical characteristics and a single receiver. We analyzed the effect of transmit diversity by fixing the transmitter at the wrist (0.5 m above the floor) and shoulder (1.5 m above the floor) of the patient body. The coordinators at the shoulder and wrist were in synchronism for the transmission and were employed to provide spatial diversity. It should be noted that the present mobility model could be designed more practically in accordance with actual hospital room environments.

### 5.2. Results and Discussion

**Figure 11:**
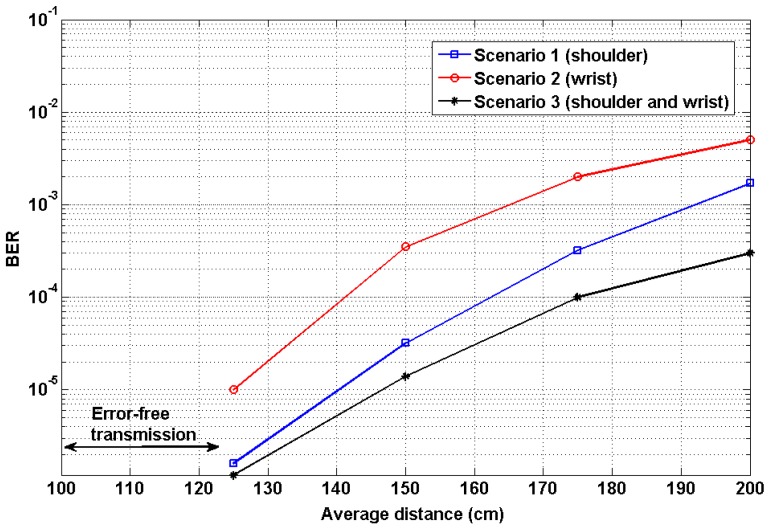
Performance analysis: BER versus transmission distance.

Figure 11 shows the experiment results of the proposed mobility scenarios expressed in terms of BER. The average transmission distance is the vertical distance measured from the chest of the patient to the PD at the ceiling. We performed several experiments by varying distances at a range of 0 to 200 cm. For a relatively short distance of up to 120 cm, the system achieved error-free transmission. The performance of the first mobility scenario achieved a BER value of 1.6 × 10^−6^ at 125 cm. The performance of the second scenario achieved a BER value of 0.1 × 10^−4^. The system performance was degraded when the distance between the transmitter and receiver increased. The performance was significantly affected if the transmission distance was greater than 1.5 m. This was because the ambient light from the environment interfered with the received signal. The ambient light was the indoor artificial lights for illumination purposes. Since the experiment was performed in a typical indoor lighting environment to emulate practical working conditions, the received signal cannot be identified easily due to the wideband ambient light. To analyze the effect of ambient light, we measured the light intensity at an accuracy level of ±0.5 lux with a sampling period of 1 s. The measured light intensities were 1000 and 200 lux at distances of 25 cm and 200 cm, respectively. The transmission distance can be increased by increasing the output power of the LED or dimming the environment. From the above two scenarios, it is clearly seen that the coordinator at the shoulder (scenario 1) shows a reliable data transmission with efficient patient mobility and hence it is found to be an optimum position for the coordinator on the patient body.

As part of further evaluation, we continued to perform experiments for the third scenario to analyze the effect of transmit diversity. Figure 11 shows the BER performance of transmit diversity. In this diversity technique, the transmission was carried out with two transmitters and a single receiver. The monitoring ECG data was transmitted from the transmitters fixed at the shoulder and wrist part of the patient body. The BER value at a distance of 120 cm was found to be 1.2 × 10^−6^. Thus, the performance of the transmit diversity outperforms mobility scenarios 1 and 2. From the results, it is apparent that the average system performance was improved. Hence, the diversity improved the overall system performances, particularly at large distances. The performance mainly depends upon the distance between the transmitter and receiver, the reflectivity of the wall surfaces, the responsivity of the receiver, the orientation of the coordinator, and indoor ambient light sources.

In the proposed work, it should be noted that we employed the adaptive threshold method to extract the received signals more precisely. It is aimed at excluding false data in the reconstructed signal. In addition, the threshold selection was strongly dependent on the ambient noise in the indoor environment. We have developed this mobility model for a single patient analysis. This idea can be further extended for the multiple-patient mobility, considering advanced interference mitigation techniques.

Table 4 compares the proposed mobility model with the conventional mobility system [11] and also shows the characteristics. It is clear that the proposed OBAN-based mobility model has advantages of high spectrum bandwidth, less complex transceiver, error-free transmission for a relatively longer distance, and reliable mobility support to the patient over conventional approaches.

## 6. Conclusions

The OBAN system with mobility support using non-directed optical wireless links has been investigated. The OBAN mobility scenario was defined by the optical coordinator on the patient body. The analytical expression for the indoor channel model incorporating multiple reflection points for a non-directed optical link was derived and validated through simulation. As a proof-of-concept, the ECG signal was employed to analyze the reliability of the proposed mobility support model. The experimental results demonstrated that the transmit diversity (scenario 3) presented very efficient user mobility performance achieving a BER value of 1.2 × 10^−6^ over a distance of 1.25 m.

## Figures and Tables

**Table 1:** Simulation parameters.

Parameters	Values
Room dimensions (x, y, z)	5 m × 5 m × 3 m
Number of LEDs	2 (1 at shoulder, 1 at wrist)
Transmitted power per light emitting diode (LED))	20 mW
Semiangle at half power	50°
Number of PD	1
Physical area of the receiver PD	1.0 cm^2^
Receiver Field of view ( FOV)	70°
Responsivity	1 A/W
Reflection coefficient	0.8
Speed of the patient	0.5 m/s
Distance between the LED (above the floor) and PD (ceiling)	1.5 m

**Table 2:** Comparative analysis of IR-OCDMA, VLC-OCDMA, and the proposed scheme.

Parameter	IR-OCDMA [11]	VLC-OCDMA	Proposed Scheme
Number of nodes	5	2	2
Code length	7200	7200	–
Room dimensions	7 m × 5 m × 2.5 m	5 m × 5 m × 3 m	5 m × 5 m × 3 m
Achievable BER ($P_{t}$ = 60 mW)	1.61 × 10^−5^	3.75 × 10^−4^	2.74 × 10^−8^

**Table 3:** Experiment parameters.

Parameters	Values
Room dimensions (x, y, z)	5 m × 5 m × 3 m
Number of LEDs	2
Optical output power of each LED	20 mW
Reflection coefficient	0.8
Physical area of the photodetector	1.0 cm^2^
Field of view (FOV)	70°
Semi angle at half power	50°
Responsivity	1 A/W
Speed of the patient	0.5 m/s

**Table 4:** Comparison of proposed system and existing system.

Specifications	IR-OWBAN [11]	Proposed OBAN
Configuration	Diffuse optical reflections	Non-directed optical links
Spectrum usage	IR spectrum	Visible spectrum
Number of LEDs (Transmitter)	3	2
Coordinator	Photodetector	LED
Receive position (PD)	Shoulder (on-body)	Ceiling
Mobility support	Yes	Yes
Speed of the patient	Not mentioned	0.5 m/s
Validation	Theoretical analysis	Simulations and experiments
Complexity	Increases with number of nodes	Not complex
